# L1 Norm based common spatial patterns decomposition for scalp EEG BCI

**DOI:** 10.1186/1475-925X-12-77

**Published:** 2013-08-06

**Authors:** Peiyang Li, Peng Xu, Rui Zhang, Lanjin Guo, Dezhong Yao

**Affiliations:** 1 School of Life Science and Technology, University of Electronic Science and Technology of China, Chengdu 610054, China

**Keywords:** Brain computer interface, Common spatial pattern, L1 norm, Motor imagery, Singular value decomposition

## Abstract

**Background:**

Brain computer interfaces (BCI) is one of the most popular branches in biomedical engineering. It aims at constructing a communication between the disabled persons and the auxiliary equipments in order to improve the patients’ life. In motor imagery (MI) based BCI, one of the popular feature extraction strategies is Common Spatial Patterns (CSP). In practical BCI situation, scalp EEG inevitably has the outlier and artifacts introduced by ocular, head motion or the loose contact of electrodes in scalp EEG recordings. Because outlier and artifacts are usually observed with large amplitude, when CSP is solved in view of L2 norm, the effect of outlier and artifacts will be exaggerated due to the imposing of square to outliers, which will finally influence the MI based BCI performance. While, L1 norm will lower the outlier effects as proved in other application fields like EEG inverse problem, face recognition, etc.

**Methods:**

In this paper, we present a new CSP implementation using the L1 norm technique, instead of the L2 norm, to solve the eigen problem for spatial filter estimation with aim to improve the robustness of CSP to outliers. To evaluate the performance of our method, we applied our method as well as the standard CSP and the regularized CSP with Tikhonov regularization (TR-CSP), on both the peer BCI dataset with simulated outliers and the dataset from the MI BCI system developed in our group. The McNemar test is used to investigate whether the difference among the three CSPs is of statistical significance.

**Results:**

The results of both the simulation and real BCI datasets consistently reveal that the proposed method has much higher classification accuracies than the conventional CSP and the TR-CSP.

**Conclusions:**

By combining L1 norm based Eigen decomposition into Common Spatial Patterns, the proposed approach can effectively improve the robustness of BCI system to EEG outliers and thus be potential for the actual MI BCI application, where outliers are inevitably introduced into EEG recordings.

## Background

Brain Computer Interface (BCI) is to establish the communication between human and some output devices such as a computer application or a neuroprosthesis, by means of noninvasive [1-3] or invasive approaches [1,4] Motor imagery based BCI uses the information correlated with amplitude modulations of sensory motor rhythms (SMR), which can reflect the motor intention of the subjects [1,5]. Because recent study reveals that even the patients diagnosed with amyotrophic lateral sclerosis (ALS) can accomplish SMR modulations [6], this kind of BCI has potential application for those patients with full or partial motor function impaired and has attracted wide attention in the fields such as rehabilitation [7], assistance of paralyzed patients’ movement [6], therapy of attention deficit hyperactivity disorder (ADHD) [8] and epilepsy [9]. Therefore, the main work in current work will focus on how to improve the performance of MI based BCI by considering the outlier effect.

The modulation of SMR will generate the event-related desynchronization (ERD) usually followed by event-related synchronization (ERS) [10]. The common spatial pattern (CSP) has been proved to be powerful to extract those motor imagery related features [11-13]. CSP algorithm aims to find directions (i.e., spatial filters) that maximize variance for one class and minimize variance for the other class at the same time [13]. Berlin BCI team has developed a robust online system (BBCI) using CSP to extract the motor imagery related features [14]. Despite of its popularity and efficiency, CSP is also known to be highly sensitive to outliers, which widely exists in the practical BCI application due to the ocular moment, head motion or the loose contact of electrodes [1]. Even a single outlier can dramatically change the subspace spanned by the generalized eigenvectors and thus severely distort the global solution of CSP, resulting in a meaningless feature. The standard CSP utilizes the L2-norm SVD to find the spatial filter, obtained by solving the generalized eigenvalue problem, which indicates that the influence of the outliers will be exaggerated due to the square property of L2 norm [15].

To improve the robustness of CSP, some schemes like the regularization, sparsity and ensemble voting were presented in [16-18]. For those existing improved CSPs, the main derivation is to build the new object function with aim to lower effect of introduced noise, which may distort the original deriving point of CSP that is essentially to maximize the power difference between two tasks. Actually, the noise in recordings will be delivered into the variance covariance matrix of CSP, which may disturb the estimation for spatial filter. CSP is usually transformed into the generalized eigen decomposition problem, and the conventional CSP will be solved using singular value decomposition (SVD) to find the spatial filters. It is known that conventional SVD is based on the L2 norm, and accordingly, it is easy to be influenced by the noise introduced into the covariance matrix. Because the outliers usually have large amplitude, it may be further exaggerated due to the square used in the L2 norm structure. Derived from the fact that L1 norm is robust to the outlier noise [15,19], in this paper we present a novel robust version of CSP by introducing L1-norm instead of L2-norm to solve the eigen problem in CSP.

## Material and methods

### Methods

#### L2 Norm based CSP

The basic idea of CSP is to find a group of spatial filters that maximize the variance of band-pass filtered EEG signals from one class [20,21], while the variance from the other class are minimized. Let *ϕ*_1_ and *ϕ*_2_ be the recordings for the two tasks, the spatial filters are the projections, which is equivalent to maximize the following function,

(1) $$J\left(w\right)=\frac{w^{T}\phi_{1}^{T}\phi_{1}w}{w^{T}\phi_{2}^{T}\phi_{2}w}=\frac{w^{T}C_{1}w}{w^{T}C_{2}w}$$

where *C*_*1*_ and *C*_*2*_ are the covariance matrix for the two tasks. Note that the scaling of the projection *w* will have no effect on the object value. Equation (1) can be transformed into a constrained optimization problem of the form:

(2) $$\left\{\begin{array}{l} \underset{w}{\arg\;\max}\;w^{T}C_{1}w\\ \mathrm{subject}\;\mathrm{to}\;w^{T}C_{2}w=1 \end{array}\right)$$

By introducing the Lagrange multiplier, the objective function can be rewritten as:

(3) $$L\left(w;\lambda \right)=w^{T}C_{1}w-\lambda \left(w^{T}C_{2}w-1\right)$$

**Figure 1:**
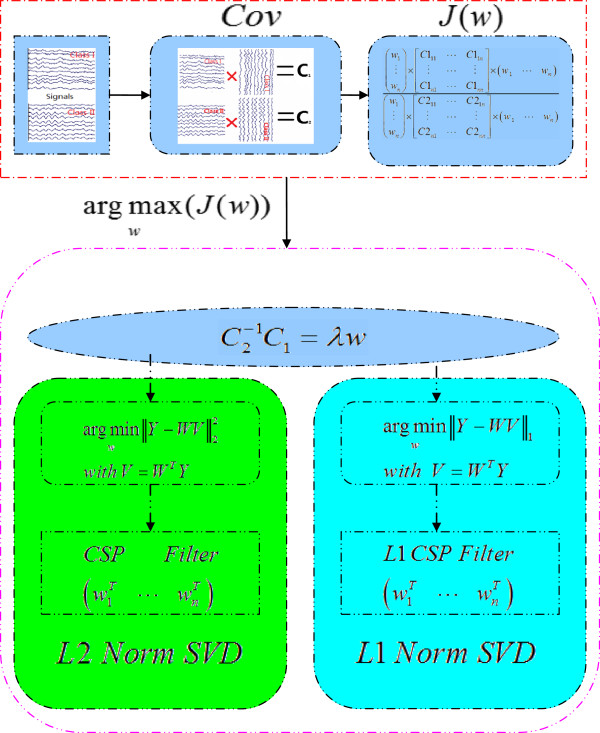
**The flow chart for the two CSPs.** The sub-procedure in the green box is for the conventional CSP, and the sub-procedure in the blue box is for the L1-SVD-CSP.

By taking the derivative of (3) respect to *w* under the condition $\frac{\partial L}{\partial w}=0$, the objective projection *w* can be estimated using the generalized eigenvalue equation,

(4) $$C_{1}w=\lambda C_{2}w$$

where *λ* denotes the eigenvalue of the generalized eigenvalue equation, and *w* is the corresponding eigen vector [3]. As for the multiple *m* spatial filters, the above equation (4) can be solved as:

(5) $$C_{2}^{-1}C_{1}W=\mathrm{\Sigma W}$$

where *W* is the matrix consisting of the eigen vectors of $C_{2}^{-1}C_{1}$, and ∑ = *diag*(*λ*_1_, *λ*_2_,......, *λ*_*m*_). The detailed implementation for CSP is shown in Figure 1.

#### L1 Norm based CSP

Obviously, the singular value decomposition can be used to find *W* in (5). Let $Y=C_{2}^{-1}C_{1}$, the objective function of L2-SVD to find the eigen vector *W* is [15]:

(6) $$\underset{W}{\arg}\;\min||Y-\mathrm{WV}|{|}_{2}^{2},\;\mathrm{with}\;V=W^{T}Y$$

Equation (6) can be obtained by the fact that the objective *Y* can be decomposed as *Y* = *WSV*^*T*^,with the right singular matrix *V* = *Y*^*T*^*WS*^–1^,and *WSV*^*T*^ = *WSS*^− 1^*W*^*T*^*Y* = *WW*^*T*^*Y*. The dual problem of the objective function can be written as [16]:

(7) $$\left\{\begin{array}{l} \underset{W}{\arg}\;\max||W^{T}Y|{|}_{2}^{2}\\ \mathrm{subject}\;\mathrm{to}\;W^{T}W=I \end{array}\right)$$

where *I* is the identity matrix. As for (7), when the conventional singular value decomposition is used to find the spatial filter, it is in essence based on the *L2* norm strategy, which will be largely influenced by the outliers delivered into the variance matrix [16].

Obviously, *L1* norm will be more immune to those outliers than *L2* norm, and we will maximize the *L1* dispersion in the feature space to estimate the projection vector *W* as presented below.

The *L1* norm SVD will have the below objective function [15]:

(8) $$\underset{W}{\arg}\;\min||Y-\mathrm{WV}|{|}_{1},\;\mathrm{with}\;V=W^{T}Y$$

Similarly, equation (8) can be transformed to below optimization problem,

(9) $$\left\{\begin{array}{l} \underset{W}{\arg}\;\max||W^{T}Y|{|}_{1}\\ \mathrm{subject}\;\mathrm{to}\;W^{T}W=I \end{array}\right)$$

Expression (9) implies to find the projection *W*, in which the projection of the original dataset will have the max dispersion in view of *L1* norm. The utilized *L1* norm based dispersion will be robust to the outlier introduced into the covariance matrix accounting for the estimation of spatial filters. Then, to solve the *L1* norm based SVD in equation (9), we adopt the fast *L1* norm iteration algorithm proposed in [15]. Obviously, the main difference between the two CSP filters is whether *L1* norm or *L2* norm based SVD decomposition is adopted for CSP filter estimation as shown in Figure 1, and the *L1* scheme may provide more robust immune ability for outliers.

### Material

#### Simulation study

##### Simulation dataset

To quantatively evaluate the performance of L1-SVD-CSP, we applied L1-SVD-CSP, the conventional CSP and the regularized CSP with Tikhonov regularization (TR-CSP) proposed in [16] to one public motor imagery EEG dataset, i.e., Dataset IVa of BCI Competition III, by adding the simulated outliers. This dataset consists of EEG signals recorded from five subjects using 118 electrodes [3,22]. In each trial, a visual cue with respect to motor imagery was shown for 3.5 s, presenting three kinds of imageries, i.e., left hand, right hand and right foot. This dataset has no outlier contained, where the trials contaminated with obvious artifacts such as ocular, head movement have been discarded by the dataset provider [23]. The tasks for the imagery of right hand and left hand are adopted to evaluate the performance in current work. As for the concerned two tasks, the total number of EEG trials for each subject was 280, and the data were band-pass filtered between 0.05 and 200 Hz and down-sampled to 100 Hz [21]. Following the reported results in [24], the time interval between 0.5 s to 2.5 s after the trial onset was selected for task recognition.

#### Simulated outliers

As for above dataset, the trials are free of such outliers due to the ocular or the imperfect contact of electrodes, and we will add some simulated outliers to construct the datasets with outlier noise corruption with aim to quantatively evaluate the methodology performance.

Considering the intrinsic characteristics of outlier that usually has extremely large amplitude, we generated the outliers from the Gaussian distribution *N*(*μ* + 10*σ*, *Σ*) for each subject, where *μ* and *σ* denoted the mean and variance of EEG across all channels for the training and testing datasets, and Σ was the corresponding covariance matrix. We added the outliers to the dataset by varying the number of outliers from *0.01(m + n)* to *0.05(m + n)* with step of *0.01(m + n)*, where the trials and time position for outliers corruption are randomly determined, with *m* and *n* being the number of trials in the training and testing sets, respectively. The above strategy used for outlier generation is mainly to simulate the actual condition that recordings may be corrupted with outliers of different probabilities during experiment.

#### Evaluation index for simulation study

For this dataset, when the occurrence of outliers is defined, the outliers are randomly added for 50 times, and L1-SVD-CSP as will as the original CSP and TR-CSP are used to extract the related features. The most discriminative 3 pairs of optimal CSP spatial filters (i.e. 6 filters) in the projection matrix are selected to transform the band pass filtered EEG signal, and the logarithm of the variance of the transformed surrogate channel EEG signal serves as the final features for task recognition. Regularized parameter of TR-CSP is determined by the 10-fold cross-validation based on training set proposed in [16].

After features are extracted for each of the 50 runs, linear discrimination analysis (LDA) free of the setup parameter optimization is used for classification based on a 5-fold cross-validation, and the averaged accuracies of the 50 repetitions are used as the index to evaluate the performance. Based on the 50 repetitions, the McNemar test [25] is used to investigate whether the difference between each pair of the three CSPs is of statistical significance.

#### Evaluations on real BCI dataset

##### Real BCI dataset

The dataset comes from the MI BCI system developed in our group, consisting of EEG data from 13 subjects. During online experiment, subjects were required to sit in a comfortable armchair in front of a computer screen, and they were asked to perform motor imagery with left hand or right hand according to the instructions appeared on the screen. Motor imagery lasts for 5 seconds, and follows a 5 seconds rest. 15 Ag/AgCl electrodes covers sensorimotor area were used to record the EEG, and the signals were sampled with 1000 Hz and band pass filtered between 0.5 Hz and 45 Hz. 4 runs on the same day were recorded for each subject, with each run consisting of 50 trials, 25 trials for each class, and there is a 3 minutes break between the two consecutive runs. All experiments were performed in accordance with the Ethical Committee of University of Electronic Science and Technology of China (UESTC). Informed consent was obtained from all participants, according to the Declaration of Helsinki.

#### Preprocessing

All the EEG segments during motor imagery are selected for analysis, and those trials with outliers caused by ocular movement, head movement are involved in further analysis, without specific inspection to remove them. The specific frequency band for each subject is obtained by [26], and then used to design band pass filter for the EEG data. The LDA classifier still used the 6-dimensional features corresponding to the most discriminative three pairs of optimal CSP spatial filters as input for task recognition.

## Results

Table 1 lists the classification accuracy of 5 subjects (aa-ay) with increasing occurrence possibility of outliers when different CSPs are used for feature extraction, where values in bold denotes the best result and *, † as well as ‡ reflect that a significant difference exists between each pair of the three methods revealed by McNemar test (p < 0.05).

**Table 1:** Classification accuracy when outlier is introduced with different occurrence rate

**Subject**	**Method**	**Frequency**				
		**0.01**	**0.02**	**0.03**	**0.04**	**0.05**
aa	CSP	0.55 ± 0.07	0.54 ± 0.08	0.54 ± 0.08	0.53 ± 0.07	0.54 ± 0.08
	TR-CSP	0.68 ± 0.07†	0.68 ± 0.07†	0.67 ± 0.07†	0.67 ± 0.07†	0.67 ± 0.07†
	L1-SVD-CSP	0.74 ± 0.08‡ *	0.73 ± 0.07‡ *	0.72 ± 0.07‡ *	0.71 ± 0.07‡ *	0.71 ± 0.08‡ *
al	CSP	0.90 ± 0.12	0.84 ± 0.14	0.83 ± 0.14	0.83 ±0.13	0.83 ± 0.14
	TR-CSP	0.94 ± 0.08†	0.91 ± 0.07†	0.89 ± 0.07†	0.87 ±0.07†	0.87 ± 0.06†
	L1-SVD-CSP	0.95 ± 0.03‡ *	0.93 ± 0.03‡ *	0.92 ± 0.07‡ *	0.92 ± 0.06‡ *	0.91 ± 0.07‡ *
av	CSP	0.59 ± 0.09	0.59 ± 0.10	0.60 ±0.09	0.60 ± 0.09	0.61 ± 0.08
	TR-CSP	0.63 ± 0.10†	0.63 ± 0.08†	0.66 ± 0.07†	0.65 ± 0.09†	0.64 ± 0.09†
	L1-SVD-CSP	0.72 ± 0.06‡ *	0.70 ± 0.06‡ *	0.71 ± 0.08‡ *	0.71 ± 0.07‡ *	0.69 ± 0.08‡ *
aw	CSP	0.65 ± 0.11	0.63 ± 0.10	0.64 ± 0.10	0.60 ± 0.11	0.61 ± 0.10
	TR-CSP	0.74 ± 0.12†	0.72 ± 0.08†	0.72 ± 0.03†	0.72 ± 0.03†	0.72 ± 0.03†
	L1-SVD-CSP	0.81 ± 0.08‡ *	0.80 ± 0.07‡ *	0.80 ± 0.07‡ *	0.77 ± 0.08‡ *	0.77 ± 0.06‡ *
ay	CSP	0.57 ± 0.09	0.64 ± 0.13	0.68 ± 0.13	0.72 ± 0.14	0.76 ± 0.14
	TR-CSP	0.84 ± 0.06†	0.83 ± 0.06†	0.82 ± 0.06†	0.82 ± 0.06†	0.82 ± 0.06†
	L1-SVD-CSP	0.93 ± 0.06‡ *	0.92 ± 0.02‡ *	0.91 ± 0.03‡ *	0.91 ± 0.04‡ *	0.90 ± 0.04‡ *

The McNemar test is performed to investigate the recognition difference between the three spatial filters, values in bold denote the better result. ‘‡’ indicates the significance between CSP and L1-SVD-CSP (p < 0.05); ‘†’ indicates the significance between CSP and TR-CSP (p < 0.05); ‘*’ indicates the significance between TR-CSP and L1-SVD-CSP (p < 0.05).

**Figure 2:**
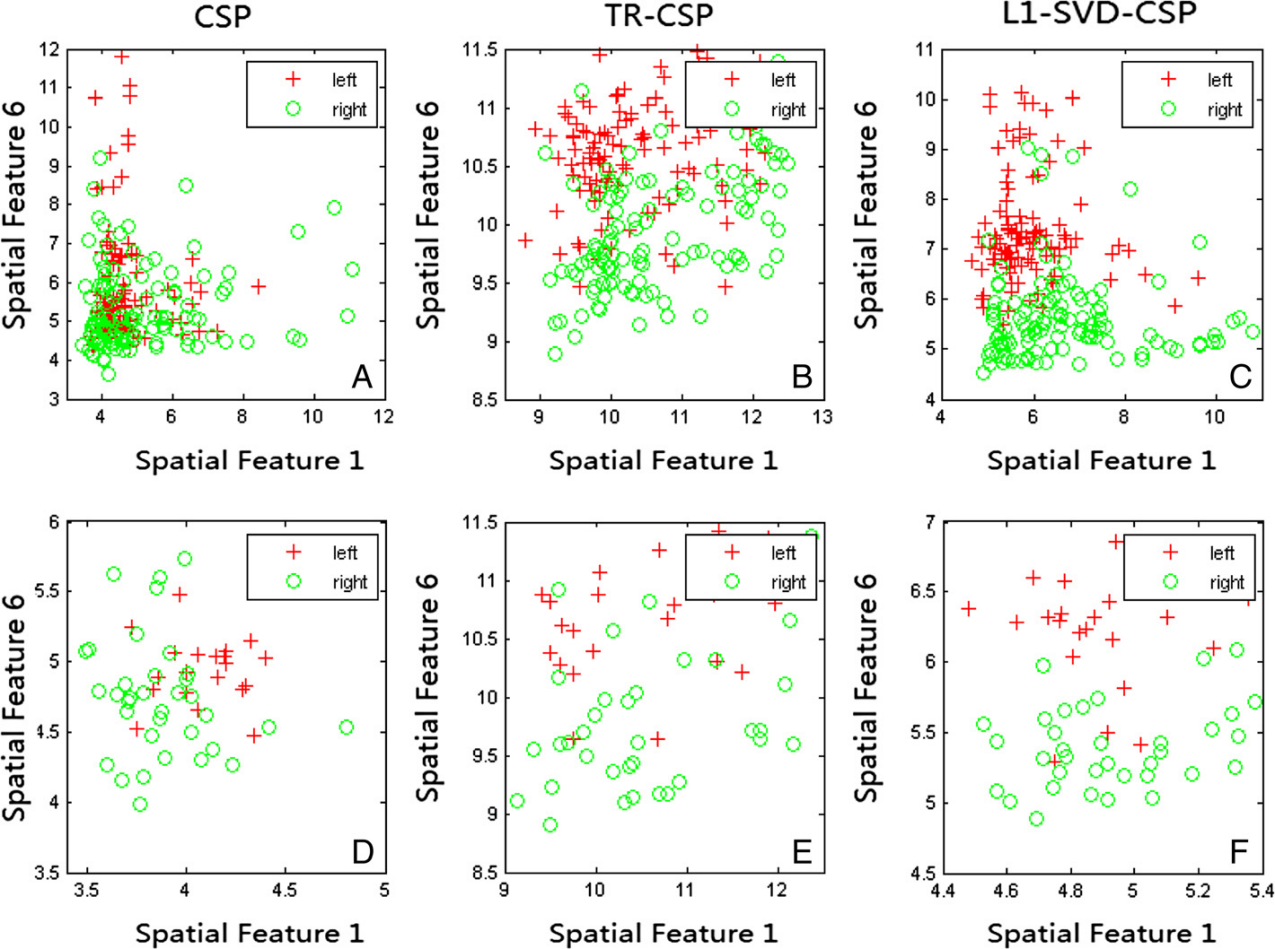
**The scatter plots of features for the two discriminative filters. (A)** The training features extracted with the conventional CSP; **(B)** The training features extracted with TR-CSP; **(C)** The training features extracted with L1-SVD-CSP; **(D)** The testing features extracted with the conventional CSP; **(E)** The testing features extracted with TR-CSP; **(F)** The testing features extracted with L1-SVD-CSP. Red“+” represents the feature of left hand imagination, and green“o”denotes the feature of right hand imagination.

**Figure 3:**
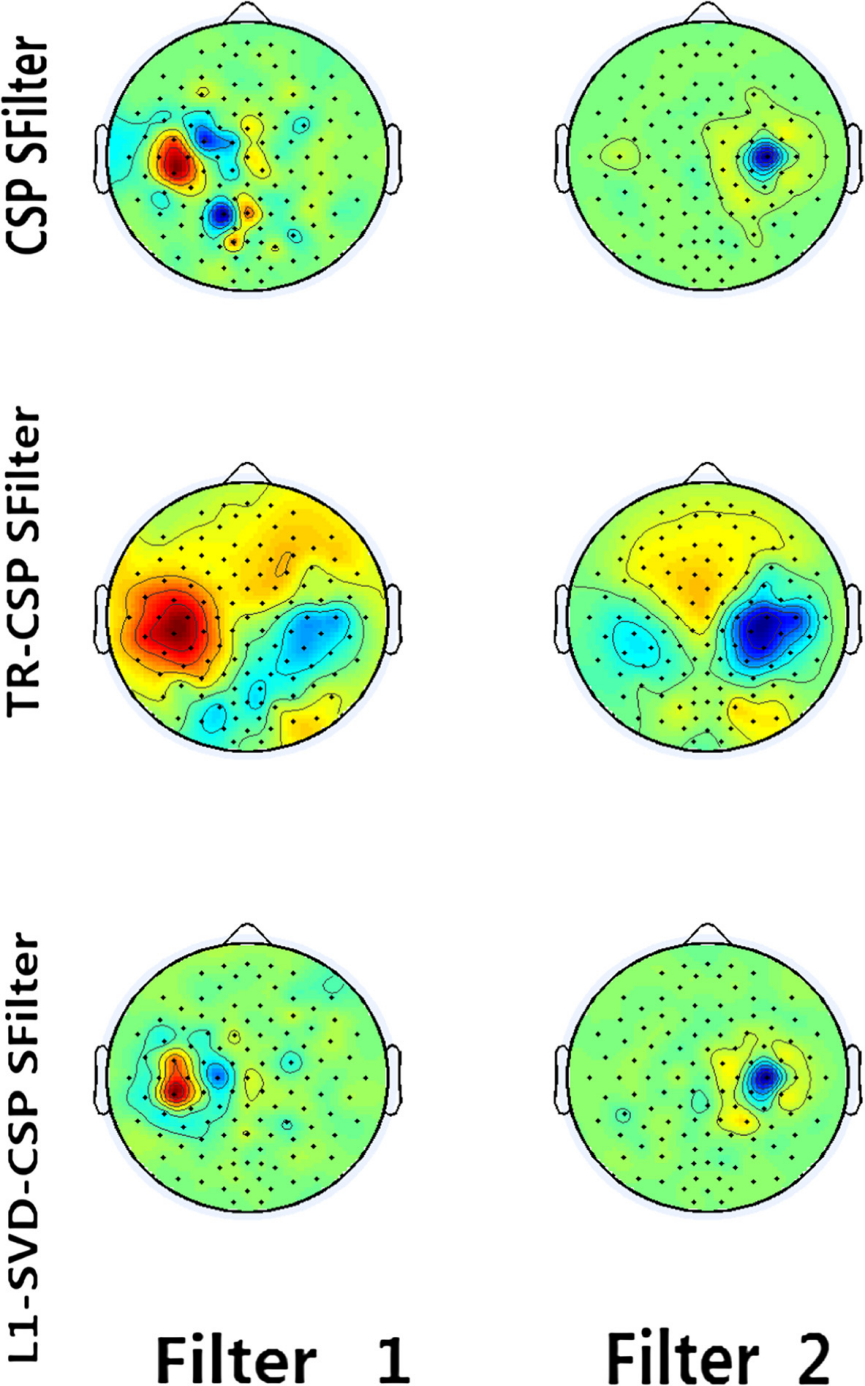
**The scalp topology of two most discriminative CSP filters learned from the train set with 0.05 outlier occurrence rate.** The first row is the two conventional CSP filters, and the second and third rows are respectively the TR-CSP filters and L1-SVD-CSP filters.

To reveal the working mechanism accounting for the difference in Table 1, we will use figures to visually show the properties of the three kinds of filters. The feature in simulation study is of 6-dimension, corresponding to the 3 largest eigenvalues and the 3 smallest eigenvalues. Figures 2 gives the scatter plots of features for the two discriminative filters determined by the largest (x-ordinate) and smallest (y-ordinate) eigenvalues for both the training and testing datasets of subject al in one of 50 runs with the occurrence rate of outliers being 0.05. The corresponding scalp topologies for the two most discriminative CSP filters in the 0.05 occurrence condition are given in Figure 3. Figures 2 and 3 consistently show the obvious difference existing among the three kinds of CSP filters from both the feature aspect and filter spatial distribution, which may account for the performance difference among the three kinds of filters revealed in Table 1.

For the real BCI dataset, the first 2 runs are utilized as training set to estimate the spatial filters by L1-SVD-CSP, CSP and TR-CSP, respectively, and the last 2 runs are used as test set. The classification accuracies obtained by the three feature extraction strategies for 13 subjects are listed in Table 2, and McNemar test is also used to investigate the difference between each pair of the three methods (p < 0.05).

**Table 2:** Classification accuracy for the real BCI dataset

**Subjects**	**Classification accuracy**		
	**CSP**	**L1-SVD-CSP**	**TR-CSP**
CXY	**0.90**	**0.90**	0.84
WZQ	0.66	**0.69**	0.66
FNX	0.66	**0.67**	**0.67**
GK	0.93	**0.94**	**0.94**
LPY	0.76	0.78	**0.79**
JSL	0.57	0.63	**0.65**
LB	0.70	**0.79**	0.70
MXY	0.61	**0.62**	0.61
SG	0.58	**0.62**	0.58
WCF	0.80	**0.86**	0.80
WH	0.59	**0.62**	0.59
XXC	0.93	**0.96**	0.95
XJP	0.98	**0.99**	0.98
WXY	0.74	**0.75**	0.71
Mean result	0.74 ± 0.14	0.77 ± 0.13‡ *	0.75 ± 0.13

The McNemar test is performed to investigate the recognition difference among the three spatial filters, values in bold denote the better result. ‘‡’ indicates the significance between CSP and L1-SVD-CSP (p < 0.05); ‘*’ indicates the significance between TR-CSP and L1-SVD-CSP (p < 0.05).

## Discussion

Typical BCI performances are largely influenced by the recording outliers due to ocular movement, head movement or the loose contact of electrodes. The motivation of this paper is to use the L1-SVD to construct a robust CSP for motor imagery related feature extraction.

The conducted simulation studies are to simulate the recordings in different time periods that may have different outliers involved. Table 1 reveals that both L1-SVD-CSP and TR-CSP have the better performance with statistical sense (p < 0.05) than the conventional CSP for all the occurrence probabilities of outliers across the five subjects, which demonstrates the effectiveness of L1-SVD-CSP and TR-CSP to suppress the outlier influence. Between L1-SVD-CSP and TR-CSP, L1-SVD-CSP shows the statistical improvement (p < 0.05) compared to TR-CSP.

Figures (2) ~ (3) visually reveal the different influences of outlier on CSP filters. For the training features extracted by the standard CSP in Figure 2(A), it is obvious that many features from class I are close to the features from class II, where many red samples are overlapped by the green samples. The overlaps between the two classes indicate that the spatial filters estimated by standard CSP are largely influenced by outliers in training set and accordingly the biased CSP filters will not project test samples into the discriminative space separately for the two classes as shown in Figure 2(D). When regularization is used, the outlier influence can be suppressed and the more samples can be visually classified as shown in Figure 2(B) and Figure 2(E) with relatively better recognition boundaries between the two classes compared to the conventional CSP. In theory, TR-CSP is to impose the L2 norm constraints on the spatial filter, which may not be competitive to deal with outlier effect though it actually can suppress the corresponding effect to some degree. However, in Figure 2(C), we can see that more discriminative features are extracted by L1-SVD-CSP with less number of samples between the two classes overlapped, indicating that the spatial filters estimated by L1-SVD-CSP are more robust to the outliers. The test features in Figure 2(F) also prove that L1-SVD-CSP filters can provide good classification information for test samples with very obvious recognition boundaries between the two classes. Moreover, the scalp topologies in Figure 3 clearly show the spatial difference among the three kinds of CSP filters. Actually, the first CSP filter in Figure 3 is to extract for the right hand MI, and the second one is for the left hand MI. The related MI area that can differentiate the left and right MI tasks has been proved to be close to the electrode C3 and C4, and the good CSP filters will provide more emphasis for the electrodes around electrode C3 and C4 with relatively larger coefficients. The first row in Figure 3 clearly shows that those electrodes close to C3 are actually emphasized by conventional CSP filters, while some electrodes obviously out of motor area are also provided with large coefficients. Essentially, those non-motor areas are undesirably introduced by outliers, and it will finally influence the feature extraction for the test samples. When L2 norm regularization is used to the spatial filters, the L2 norm will smooth the filters and the enlarged and blurring spatial distribution [27] will be estimated as shown in the middle row of Figure 3. Accordingly, the smoothed spatial distribution will involve other unexpected electrodes out of motor area for feature extraction. Interestingly, when L1-SVD-CSP is adopted, those artifacts effects out of motor area are effectively compressed, resulting a clear filter with emphasis mainly on the expected motor areas close to C3. It is the spatial difference of CSP filters that determines the performance difference among these CSP filters.

As for the actual BCI dataset, the outliers are not visually removed as the BCI competition dataset. Similar to the results for the simulated dataset by adding additional outliers, L1-SVD-CSP also shows better performance than both the conventional CSP and TR-CSP with approximately 3% improvement, and the McNemar test also revealed that the accuracy improvement evaluated on those 13 subjects is of statistical sense (p < 0.05).

The improvement brought by L1-SVD-CSP is mainly due to the utilization of L1-SVD for spatial filter estimation, which is less prone to the presence of outliers with large amplitude. By contrast, in the conventional CSP method, the features for classification were obtained through the L2-norm eigen decomposition, which exaggerated the effect of outliers by imposing square operation to signals. The utilization of L2 norm constraint on spatial filter estimation in TR-CSP will smooth and blur the spatial filters, which may involve other uncorrelated electrodes information into the extraction of the motor rhythm related features. When L1-SVD-CSP utilized the L1-norm SVD to estimate the eigenvector, the effect of outliers will be suppressed as proved in other reported studies [15].

In current version, L1 norm is used to re-format the solving procedure of CSP. Usually, the L1 problem is of larger complexity compared to the L2 based problem, which may be not suitable for online BCI training. In this paper, we adopted the fast iteration algorithm proposed in [16] to solve the SVD problem, and it can dramatically reduce the training time. However, L1 norm is the approximation to L0 norm, and it may lower the sparsity to some degree compared to L0 norm [28]. In the future, we will also use the more efficient sparse measurements like L1/2 or L0 norms to further improve the performance for CSP feature extraction.

In practice, the specifically designed classifier can also be used to effectively suppress the outlier effect. In Lei et al. 2009 [17], the authors utilizes the Bayesian framework to combine the feature extraction and classification together, which can automatically select the reliable features and abandon those artifact influenced features for final classification. Inspired by this scheme, in our future work, we will combine our sparse feature extraction approach with the Bayesian framework to suppress outlier effect both for feature extraction and classification, which may result in more promising technique for practical BCI system.

## Conclusions

In the actual BCI application situation, the outliers are inevitable and it needs to tackle the outlier effect. The conducted comparison on both the simulated datasets and the real BCI dataset prove that L1-SVD-CSP can effectively lower the outlier effects to extract more reliable MI features for BCI task recognition.

## Abbreviations

BCI: Brain computer interfaces; CSP: Common spatial patterns; EEG: Scalp-recorded electroencephalogram; SMR: Sensory motor rhythms; ALS: Amyotrophic lateral sclerosis; ADHD: Attention deficit hyperactivity disorder; ERD: Event-related desynchronization; ERS: Event-related synchronization; SVD: Singular value decomposition; TR-CSP: The regularized CSP with Tikhonov regularization.

## Competing interests

The authors declare that they have no competing interests.

## Authors’ contributions

PYL and PX designed the concept, analyzed the data; PYL, PX and DZY wrote the manuscript; LJG and RZ recruited the subjects, and performed the experiments. All authors read and approved the final manuscript.
